# Exploring the antimicrobial peptidome of nematodes through phylum-spanning *in silico* analyses highlights novel opportunities for pathogen control

**DOI:** 10.1371/journal.pntd.0011618

**Published:** 2023-09-06

**Authors:** Allister Irvine, Sharon A. Huws, Louise E. Atkinson, Angela Mousley

**Affiliations:** Microbes & Pathogen Biology, The Institute for Global Food Security, School of Biological Sciences, Queen’s University Belfast, Belfast, United Kingdom; Institute of Parasitology,Biology Centre, AVCR, CZECH REPUBLIC

## Abstract

Antimicrobial Peptides (AMPs) are key constituents of the invertebrate innate immune system and provide critical protection against microbial threat. Nematodes display diverse life strategies where they are exposed to heterogenous, microbe rich, environments highlighting their need for an innate immune system. Within the Ecdysozoa, arthropod AMPs have been well characterised, however nematode-derived AMP knowledge is limited. In this study the distribution and abundance of putative AMP-encoding genes was examined in 134 nematode genomes providing the most comprehensive profile of AMP candidates within phylum Nematoda. Through genome and transcriptome analyses we reveal that phylum Nematoda is a rich source of putative AMP diversity and demonstrate (i) putative AMP group profiles that are influenced by nematode lifestyle where free-living nematodes appear to display enriched putative AMP profiles relative to parasitic species; (ii) major differences in the putative AMP profiles between nematode clades where Clade 9/V and 10/IV species possess expanded putative AMP repertoires; (iii) AMP groups with highly restricted profiles (e.g. Cecropins and Diapausins) and others [e.g. Nemapores and Glycine Rich Secreted Peptides (GRSPs)] which are more widely distributed; (iv) complexity in the distribution and abundance of CSαβ subgroup members; and (v) that putative AMPs are expressed in host-facing life stages and biofluids of key nematode parasites. These data indicate that phylum Nematoda displays diversity in putative AMPs and underscores the need for functional characterisation to reveal their role and importance to nematode biology and host-nematode-microbiome interactions.

## Author summary

Nematodes include some of the most abundant animals on Earth, found in almost every natural habitat. Globally, parasitic nematodes cause major impacts to human, animal and plant health. Little is known about how parasitic nematodes interact with microbes despite the microbe-rich environments that many of them inhabit. In other invertebrate animals, Antimicrobial Peptides (AMPs) are critical in defense against pathogenic microbes. By characterising nematode AMP diversity and exploring the role of nematode-derived AMPs we could reveal novel targets for antimicrobial and anthelmintic development.

In this study we profiled the distribution of five known AMP groups across the nematode phylum. This homology-directed approach demonstrated that, nematodes appear rich in AMP diversity with major differences in the abundance and distribution of putative AMP-encoding genes across nematode lifestyles and clades. In addition, transcriptomic and proteomic analyses show that many of the identified AMP-encoding genes appear biologically relevant in nematode life stages that interact with the host. This study provides a database of potential nematode-derived AMPs which can drive functional analyses to explore the role of nematode AMPs.

## Introduction

Antimicrobial Peptides (AMPs) are a group of host defence peptides that act as natural immune effectors and are present in all classes of life [1]. Invertebrates, which lack an adaptive immune system, rely on facets of innate immunity, including AMPs, to protect against microbial threat [2]. Invertebrate AMPs form the first line of defence against invading pathogens including bacteria, fungi and viruses by exerting broad-spectrum antimicrobial activities. Membranolytic AMPs are generally cationic amphipathic peptides which, through various interaction models [carpet, barrel-stave and toroidal pore; [3], bind to microbial cell membranes leading to cell lysis [4]. AMPs also stimulate invertebrate immune regulation, enhancing the endogenous immune response to protect against microbial threat [5]. Whilst AMPs have gained significant attention as novel, resistance-breaking, antimicrobial agents [6], their key roles in invertebrate innate immunity highlight an unexplored opportunity for the exploitation of AMPs as novel targets for the control of invertebrate pathogens, including nematode parasites. Phylum Nematoda belong to the clade Ecdysozoa which also includes arthropods and tardigrades [7]. Current understanding of ecdysozoan AMP function is primarily derived from arthropods [8] where they have been shown to be critical in defense against pathogens [9–11], however knowledge of the diversity, role and importance of AMPs, including in nematode parasites, is limited [12]. Harnessing nematode-derived AMPs for parasite control demands a comprehensive understanding of AMP biology which is reliant on the characterisation of AMP diversity in key nematode pathogens.

Parasitic nematodes frequently reside in hazardous host environments such as the microbe-rich intestine where they survive in close association with a broad range of microorganisms. Consequently, gastrointestinal nematodes are likely to require a raft of endogenous AMPs for survival within the host environment. Knowledge of AMPs in nematodes has been derived from research using the model organism *Caenorhabditis elegans* and the gastrointestinal pig parasite *Ascaris suum*, and more recently broader *in silico* studies, where five distinct AMP groups have been identified: Cecropins, Cysteine stabilised α-helix and β-sheet fold (CSαβ) peptides, Nemapores, Diapausins, and Glycine Rich Secreted Peptides (GRSPs) [13–21]. Each of these AMP groups are characterised by distinct sequence features, where the CSαβ peptides, Nemapores and Diapausins are defined by cysteine motifs [22–24] and the Cecropins and GRSPs are linear peptides defined by a specific signature sequence [23] and an enrichment of glycine residues respectively [20]. The ability to uncover AMPs in nematodes has been significantly enhanced by the availability of helminth genome and transcriptome datasets [25]. Indeed, prior to this study, the most complete profile of putative AMPs in nematodes was conducted by Tarr [19], where draft genomes and Expressed Sequence Tag (EST) datasets were examined in 58 nematode species for a sub-set of AMP groups (Cecropin, CSαβ [previously named Defensin], Nemapore). A constraint, noted by Tarr [19], was the lack of publicly available nematode ‘omics data which prevented the comprehensive characterisation of nematode AMP groups. Recent expansions and quality improvements in nematode ‘omics data present a timely opportunity for the *in-silico* characterisation of pan-phylum nematode AMP diversity that will provide a springboard to functional biology and therapeutic exploitation.

This study examines 134 nematode genomes, representing 109 species, to profile five AMP-encoding gene groups (Cecropin, CSαβ, Diapausin, Nemapore, GRSP) using a BLAST/HMM-directed bioinformatics approach. To our knowledge, the data presented here represent the most comprehensive profile of putative nematode AMPs to date and signify a critical advance in our understanding of the potential nematode AMP repertoire. These data expose nematodes as a rich source of putative AMP diversity that displays both conservation and variation within and between nematode clades, lifestyles and life stages likely reflecting biologically relevant trends.

These data reveal putative AMP diversity within phylum Nematoda and provide a database of potential AMPs that can be used to explore AMP biology in therapeutically relevant parasites to underpin novel nematode control approaches. In addition, this study has provided a novel library of potential nematode-derived natural antimicrobials which may exhibit desirable characteristics for future antimicrobial discovery.

## Materials and methods

### Identification of putative AMP-encoding genes in phylum Nematoda

**Fig 1:**
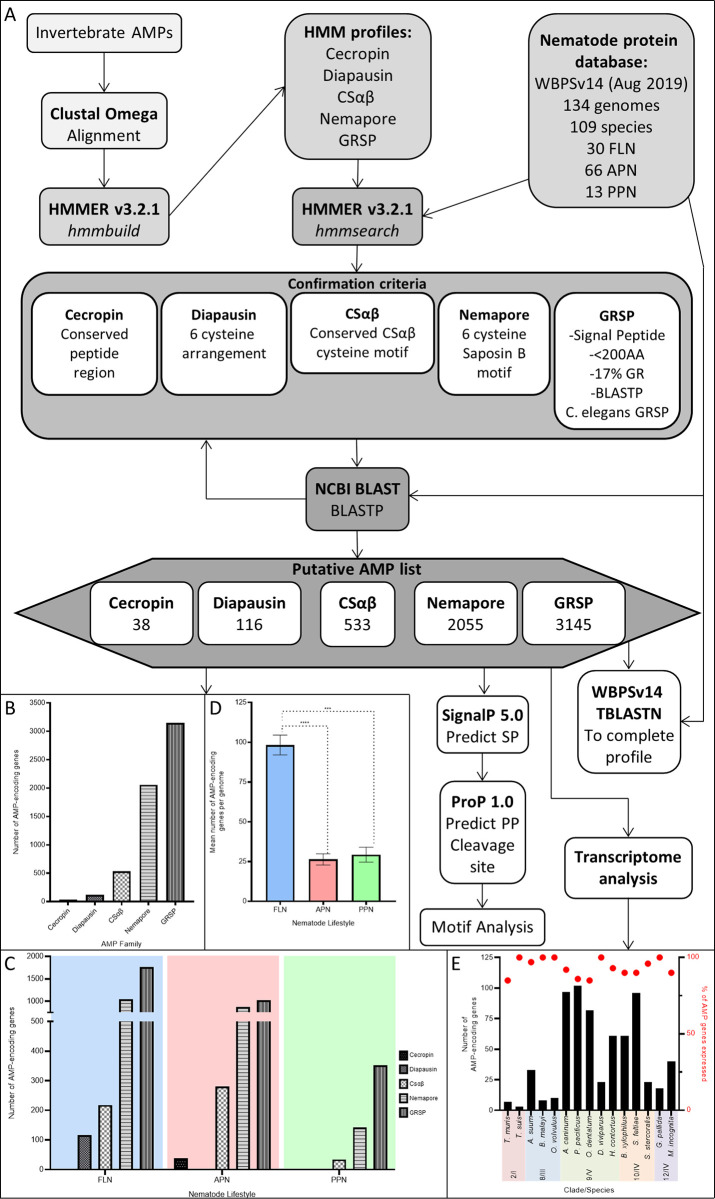
An *in silico* AMP discovery pipeline reveals that phylum Nematoda is a rich source of putative AMPs. A) Summary of BLAST/HMM-based methods for putative AMP identification. B) Number of putative AMP-encoding genes identified for each nematode AMP group. C) Total number of putative AMP-encoding genes for each AMP group across nematode lifestyles. D) Mean number of putative AMP-encoding genes per genome across each nematode lifestyle. E) Summary of putative AMP-gene expression (>2 TPM) relative to the number of putative AMP-encoding genes for key nematode species. Error bars indicate standard error of the mean. *** P<0.001; **** P<0.0001. AA, Amino Acid; AMP, Antimicrobial Peptide; APN, Animal Parasitic Nematode; BLAST, Basic Local Alignment Search Tool; BLASTP, Protein BLAST; CSαβ, Cysteine-Stabilised Alpha Beta peptide; FLN, Free-Living Nematode; GR, Glycine Rich; GRSP, Glycine Rich Secreted Peptide; HMM, Hidden Markov Model; PP; Propeptide; PPN, Plant Parasitic Nematode; SP, Signal Peptide; TBLASTN, Translated nucleotide BLAST; WBPSv14, WormBase ParaSite Version 14.

A summary of the methods in this study have been provided graphically (see Fig 1A). Genes encoding putative AMPs representing five groups (Cecropin, Diapausin, CSαβ, Nemapore and GRSP-see Table 1) were identified based on sequence similarity to the five previously identified nematode AMP groups using Hidden Markov Model (HMM) and Basic Local Alignment Search Tool (BLAST) searches. Each of these AMP groups are represented by distinct sequence features (see Table 1). HMM profiles were built using HMMER v3.2.1 (www.hmmer.org) for each AMP group and, where appropriate, for individual subgroups. Previously identified AMPs (see S1 File) were aligned using CLUSTAL Omega with default settings [26], followed by *hmmbuild* (with default settings) to construct specific AMP group HMM profiles. Where required, alignments were manually adjusted to align AMP motifs.

**Table 1:** Summary of five known nematode AMP group characteristics.

Nematode AMP Group	Peptide characteristics	Example member
Cecropin	Amphipathic α-helical structure [27]	*Ascaris suum* Cecropin P1 [28]
Diapausin	Two α-helices and a triple-stranded β-sheet structure stabilised by six cysteine motif distinct to that of CSαβ peptides [29]	*Manduca sexta* Diapausin-1 [30]
CSαβ	α-helix and two β sheet structure stabilised by at least six cysteines which form three disulfide bonds [24]	*Ascaris suum* ASABF-α [31]
Nemapore	Saposin-like fold consisting of amphipathic α-helices, strengthened by three disulfide bonds [32]	*Caenorhabditis elegans* SPP-3 [33]
GRSP	Secreted peptides with a mature peptide glycine content between 17%-74% [20]	*Caenorhabditis elegans* NLP-31 [17]

A database of nematode predicted proteins was collated from the predicted protein datasets derived from 134 nematode genomes [WormBase ParaSite V14; see S2 File;] [34–35]. Constructed HMM models were subsequently searched against the concatenated nematode predicted protein database using the *hmmsearch* command. The results of each HMM search were cross-checked to ensure that the query sequences used to build the model scored highly. This confirmed that the HMM profile successfully identified the query sequences and was specific for each AMP group. For all AMP groups, except the GRSPs, search hits with an E value <0.01 were manually assessed for high peptide similarity and conserved AMP group motifs. Where a sequence appeared incomplete, as a result of an assembly or annotation error (i.e., missing an exon or incorrectly predicted as a longer protein), the output was retained to provide a comprehensive profile of putative AMP-encoding genes. For the Cecropin group, new hits were included based only on similarity to the query sequences, whereas for the CSαβ, Diapausin and Nemapore groups hits were included based on the presence of specific conserved cysteine motifs. Note that for the Nemapores, any sequences with additional protein domains (checked using InterPro; https://www.ebi.ac.uk/interpro/) were excluded from the study to ensure that Saposin-like proteins (SAPLIPs) with likely other functions [36] were not included. As the GRSPs are characterised by repetitive glycine-rich repeats and lack other conserved motifs, individual HMMs were constructed based on the 17 GRSP subgroups identified in *C*. *elegans* and *Caenorhabditis briggsae* GRSPs [20]. Due to the highly repetitive glycine-rich nature of the GRSPs, all hits from these HMMs up to an E value of 10 were assessed and were confirmed based on GRSP criteria established previously [20]. As a result, putative GRSP hits were only considered to be authentic if they were ≤200 amino acids, contained a putative signal peptide sequence, and possessed mature peptide glycine content >17%. Despite the construction of 17 subgroup HMM profiles for the GRSPs, results overlapped making classification of putative GRSPs according to the pre-defined *Caenorhabditis* GRSP subgroups difficult. Consequently, GRSPs hits were not classified into discrete subgroups in this study.

Post HMM analyses, all HMM-derived putative AMPs were employed as BLASTp query sequences in the NCBI command line BLAST application (version 2.9.0, April 2019) against the concatenated nematode predicted protein database constructed previously. BLASTp returns were confirmed using the same criteria as above for each AMP group and new confirmed hits were repeatedly used as BLASTp queries until no new results were retrieved. Due to the high number of putative GRSP hits returned by HMM and BLASTp searches and the potential for false-positive returns, all putative GRSP hits were subjected to an additional BLASTp search against the *C*. *elegans* protein database on the WormBase ParaSite BLAST server (with the low complexity filter off; Version 14); where a GRSP identified previously [20] was not returned within the top five hits of the additional BLASTp search (sorted by E value) the original putative GRSP hit was removed from further analyses.

Putative AMP prepropeptides were analysed for the presence of signal peptide sequences and propeptide cleavage sites using SignalP 5.0 [standard Sec/SPI settings, Eukarya organism; [37] and ProP 1.0 [default furin-specific prediction; [38] respectively. For complete sequences, putative mature peptides (based on the removal of predicted signal peptide and propeptide regions) were submitted to the Antimicrobial Peptide Scanner [APS; model- vr.2 Feb2020; 39] to assess antimicrobial potential.

The CSαβ group was subsequently classified into subgroups (Mollusc/Nematode Defensin, Traditional Insect Defensin, Macin and Drosomycin) based on number of cysteines and their arrangement according to the cysteine reference array established previously [24].

To reduce the possibility of false-negative returns post BLASTp, tBLASTn searches using query sequences from closely related species with a positive hit were employed (via WormBase ParaSite V14 BLAST server); this approach confirmed null returns and checked for the presence of unannotated sequences. Where an unannotated putative AMP was identified, the nematode AMP profile was updated to reflect this; however unannotated genes could not be included in relative abundance or transcriptome analyses. Due to high sequence variability and gene expansions in specific genera it was not possible to accurately assign AMP genes as orthologs.

### Clustered analysis of sequences (CLANS) analysis

Sequence similarity between identified AMP candidate groups was visualised using the Clustered Analysis of Sequences (CLANS) Java application [40]. For all putative AMP groups except the GRSPs, a FASTA file of full-length complete sequences were submitted to the CLANS web-utility (https://toolkit.tuebingen.mpg.de/tools/clans) in the MPI Bioinformatics Toolkit [41–42] to generate all-against-all pairwise BLASTP searches using the BLOSUM62 scoring matrix. Sequence similarity networks were visualised in two dimensions using the CLANS Java application at the most appropriate E-value limit to facilitate cluster separation after 10,000 clustering rounds. The CLANS network was manually coloured and annotated to reflect group and or subgroup classification.

### Transcriptome analyses of putative AMP-encoding genes

Publicly available life-stage specific RNAseq datasets for 15 nematode species, representing a range of nematode clades and lifestyles, were retrieved from WormBase ParaSite v14 (see S2 File). A threshold of ≥2 Transcripts Per Million (TPM) was employed as a cut-off for expression [43]. For heatmap construction TPM values (TPM +1) were Log2 transformed and converted to Z values using Heatmapper [www.heatmapper.ca/; [44]. Heatmaps were scaled by row and genes were clustered by row using the Average Linkage method with Euclidean Distance Measurement method.

### Statistical analyses

All graphs and statistical analyses were produced using GraphPad Prism 9 (www.graphpad.com/). Data were tested for normality using the Kolmogorov-Smirnov test [45]. Non-normally distributed data were tested for significance using Krustal Wallis Test with Dunn’s multiple comparisons test [46].

## Results and discussion

### Phylum Nematoda is a rich source of putative antimicrobial peptides

**Fig 2:**
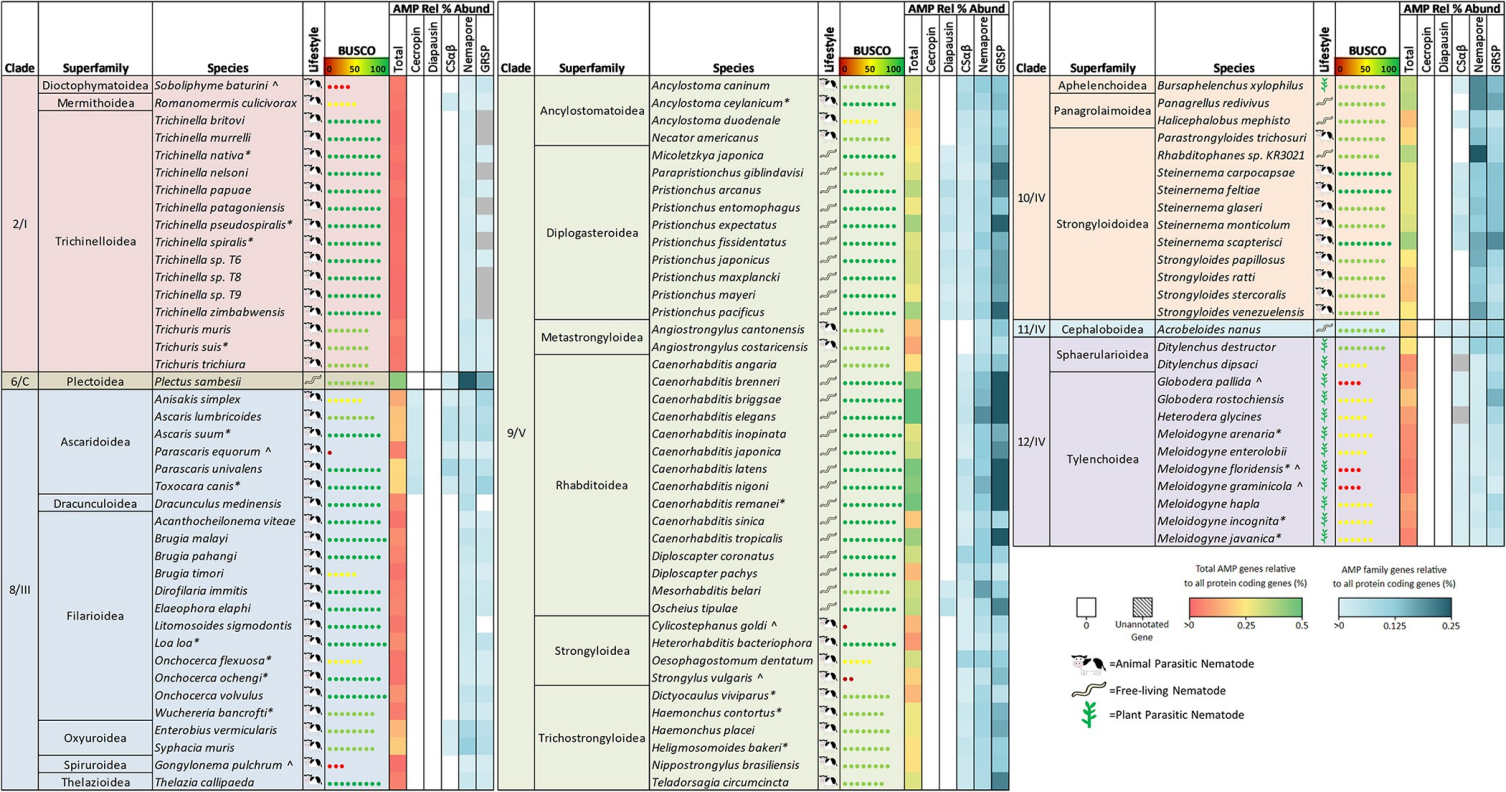
Relative abundance of putative AMP genes reveal AMP profile diversity across nematode clades and lifestyles. Nematode species (109 nematode species; 134 genomes) are organised by clade [47–48] and superfamily designation (NCBI taxonomy browser; https://www.ncbi.nlm.nih.gov/taxonomy). Relative abundance was calculated as a percentage of putative AMP genes relative to total protein coding genes per genome. BUSCO complete scores were obtained from WormBase ParaSite V14 [https://parasite.wormbase.org/index.html] [34]. ^ indicates a genome with <50 BUSCO complete score. Asterisk indicates multiple genome assemblies were used.

To investigate putative AMP abundance and diversity across phylum Nematoda, five known nematode AMP groups [Cecropin, Diapausin, CSαβ, Nemapore and GRSP] [19–21] were profiled in 109 nematode species (134 genomes; representing 7 clades, 30 free-living nematode (FLN), 66 animal parasitic nematode (APN) and 13 plant parasitic nematode (PPN) species; see S2 File). Our analyses identified 5887 putative AMP-encoding genes, of which >90% have not been previously reported, representing all known nematode AMP groups [38 (0.65%) Cecropin-encoding genes, 116 (1.97%) Diapausin-encoding genes, 533 (9.05%) CSαβ-encoding genes, 2055 (34.91%) Nemapore-encoding genes and 3145 (53.42%) GRSP-encoding genes; see Fig 1B and S3 File]. CLANS analyses of all nematode AMP groups except the GRSPs largely supported the designation of these sequences within each group and/or subgroup (see S1 Fig). Beyond the identified genes displaying sequence homology to existing nematode AMPs, a high proportion of the predicted mature peptides from complete AMP candidates were also predicted to have antimicrobial activity by the APS. Indeed, 90% of putative Cecropin peptides, 96% of putative Diapausin peptides, 99% of putative CSαβ peptides, 81% of putative Nemapore peptides and 93% of putative GRSPs achieved a prediction probability >0.5 (see S3 File) supporting their designation as AMP candidates. Whilst the relative abundance and profile of putative AMP-encoding genes vary across the phylum (see Fig 2) the representation of putative AMPs in every nematode species examined underscores the potential importance of AMPs to nematode biology. These data provide the most comprehensive profile of AMP candidates across the phylum Nematoda and demonstrate that nematodes appear AMP-rich. As these putative AMPs were identified through *in-silico* methods and share conserved sequences with experimentally validated AMPs it is important to note that these genes require functional characterisation to identify those with conserved AMP activities.

### AMP profile, abundance, and group diversity vary across nematode lifestyles

Our analyses reveal that putative AMP group profiles vary across nematode lifestyles (see Fig 1C). FLN and APN species display the greatest AMP group diversity (Diapausin, CSαβ, Nemapore and GRSP groups represented in FLNs; Cecropin, CSαβ, Nemapore and GRSP groups represented in APNs), however PPNs lack both Diapausin and Cecropin groups.

FLNs also possess the greatest number of putative AMP-encoding genes (98.31 ± 6.23 genes per genome) relative to both APNs (26.37 ±3.53 genes per genome; p<0.0001) and PPNs (29.33±4.67 genes per genome; p = 0.0001) (see Fig 1D). The higher abundance of putative AMP-encoding genes in FLNs may reflect the diversity in the terrestrial and marine environments that FLNs inhabit, and the potential heterogeneity in microbes that they are likely to encounter. Parasitic nematodes, in contrast, live endogenously within animals and plants and could benefit from host immunity which may somewhat protect against microbial threat reducing the reliance on AMPs. Parasitic nematodes, however, also likely possess an AMP arsenal which is more specialised to host-dwelling microbes and pathogens. This may also suggest that parasitic nematodes possess distinct AMP groups which have yet to be identified and require novel approaches to AMP discovery.

### Clade 9/V and 10/IV nematodes are AMP-rich

There was considerable variability in the distribution and abundance of putative AMP groups across nematode clades (see Fig 2). For clade comparisons Clades 6/C and 11/IV were excluded as there is only a single species with a publicly available genome representing each of these clades [*Plectus sambesii* (Clade 6/C); *Acrobeloides nanus* (Clade 11/IV)]. Both of these species are free-living and display high putative AMP diversity (see Fig 2); expansion of genome datasets is required to determine whether the diverse AMP profiles noted here are conserved traits for Clade 6/C and Clade 11/IV species.

Clade 9/V species displayed the highest putative AMP diversity across the phylum (80.77 ±5.74 AMP-encoding genes per genome). To account for potential differences in comparisons between genomes with different gene numbers, the number of AMP genes was calculated relative to the total number of protein coding genes (PCGs) for each genome (see Fig 2 and S4 File). By this measure, Clade 9/V displayed the highest relative abundance of putative AMP genes (average 0.31 ±0.02% per genome). Within Clade 9/V, there were differences between the relative abundance of putative AMP genes across species where *C*. *elegans* had the highest AMP complement (0.82% of total number of PCGs). In contrast, the lowest relative abundance of putative AMP genes was noted for the entomopathogenic nematode (EPN), *Heterohabditis bacteriophora*, where only 0.09% of PCGs encode AMP genes. The high abundance of putative AMP genes in *C*. *elegans* was unsurprising given that this species has provided the blueprint for nematode AMP research. Indeed, most of the query sequences used to identify AMP genes in this study originated from *C*. *elegans*. The reduced relative abundance of putative AMP genes in *H*. *bacteriophora* may be related to its symbiotic relationship with gram negative *Photorhabdus* bacteria [49]. *Photorhabdus* spp. are key to the lifecycle of *H*. *bacteriophora* and produce a range of toxins which kill the arthropod host [50]. Recently, an antibiotic with potent activity against gram negative bacteria was discovered in *Photorhabdus* [51]. It is therefore possible that *H*. *bacteriophora* benefits from AMPs produced by the bacterial symbiont reflecting the lower abundance of endogenous nematode AMPs in this species.

Beyond the AMP-rich *Caenorhabditis* genera, other genera within Clade 9/V also appear to display enriched putative AMP profiles. For example, free-living *Pristionchus* species have an enriched AMP profile with all but one species having >100 putative AMP genes (0.33 ±0.02% of all PCGs). *Pristionchus* feed on rotting plant matter and share mostly phoretic, and occasionally necromenic, relationships with arthropods [52]. It may be the case that *Pristionchus* nematodes require an enriched arsenal of AMPs, to combat the microbe-rich environments they inhabit.

The Ancylostomatoidea and Trichostrongyloidea superfamilies, which include mammalian parasites, also have a higher putative AMP abundance than the other parasitic superfamilies, e.g. the Metastrongyloidea and Strongyloidea, in this clade. Many of these parasitic species that display higher putative AMP abundance, including *Necator americanus*, *Teladorsagia circumcincta* and *Haemonchus contortus*, are known to alter the host gut microbial composition during infection [53–55]. Whilst it is currently unclear whether the putative AMPs identified here are secreted and/or excreted into the host environment and may contribute to changes in the microbial composition, nematode-derived AMPs, such as *A*. *suum* Cecropin P1, have been isolated from the host gut [13,16]. Future research should aim to establish whether excreted/secreted nematode AMPs are active against host microbiota and contribute to changes in the host microbiome during infection.

Clade 10/IV also appear to be AMP-rich (64.79 ±9.51 AMP-encoding genes per genome; 0.28 ±0.02% of PCGs in the Clade 10/IV species examined in this study encode putative AMPs). Despite the diversity in lifestyles, most Clade 10/IV species share similar numbers of putative AMPs. *Steinernema* spp. appear to have an elevated putative AMP abundance relative to other Strongyloidoidea superfamily members such as *Strongyloides* spp. Interestingly, like Clade 9/V *H*. *bacteriophora*, *Steinernema* spp. are also EPNs that display endosymbiotic relationships with gram negative bacteria. *Steinernema* spp. harbour *Xenorhabdus* bacteria which are critical for nematode establishment and ultimately kill the insect host [56]. Consistent with the relationship between *Photorhabdus* and *H*. *bacteriophora*, *Xenorhabdus* also produces antimicrobials which aid establishment and maintenance of the nematode parasites in the host [57]. *Steinernema* spp. however do not appear to have the same reduction in putative AMP gene abundance as was observed in *H*. *bacteriophora*. At present, little is known about the role that nematode-derived AMPs play in the lifecycle of EPNs.

### Clade 2/I, 8/III and 12/IV nematodes possess less diverse AMP profiles

Multiple nematode clades possess a limited putative AMP profile in comparison to Clade 9/V and 10/IV in terms of the number of AMP genes and the distribution of AMP groups. These more limited putative AMP profiles do not appear to be a result of poorer genome quality as many of the genomes with less diverse putative AMP profiles display genome BUSCO quality scores >90. Clade 2/I nematodes possess the most limited putative AMP profile of all the nematode clades profiled in this study (3.44 ±0.50 AMP-encoding genes per genome; 0.02 ±0.01% of PCGs encoding AMP genes). All species from this clade possess genes encoding Nemapores and GRSPs with only *Romanomermis culicivorax* possessing CSαβ-encoding genes (see Fig 2). Clade 2/I nematodes are generally considered to be more basal and have more limited gene complements for other protein families including neuropeptides, antioxidant enzymes, G-protein-coupled receptors (GPCRs) and ATP-binding cassette transporters (ABC transporters) [58–61]. The reduced AMP arsenal observed in Clade 2/I may indicate that elevated AMP diversity in the Crown Clades (Clade 8–12) originated as a result of gene duplications and/or selective pressures that are not observed in the basal clades. It is interesting that many Clade 2/I nematodes share similar host niches with some of the parasitic species that have more diverse AMP profiles. Indeed, both *Trichinella* and *Trichuris* species spend much of their lifecycles in the microbe-rich gut environment of their hosts. The reduced number of putative AMP-encoding genes in Clade 2/I nematodes poses an intriguing question about how these nematodes combat microbial threats without the range of AMPs that other intestinal parasitic nematodes possess. It is possible that Clade 2/I species may possess divergent AMPs or larger antimicrobial proteins that cannot be identified using the BLAST/HMM-based pipeline employed in this study.

With the exception of the Ascaridoidea (31.38 ±4.14 AMP-encoding genes per genome; 0.17 ±0.02% of PCGs encoding AMPs) and Oxyuroidea (20.50± 0.50 AMP-encoding genes per genome; 0.17 ± 0.02% of PCGs encoding AMPs) superfamilies, Clade 8/III nematodes also broadly displayed a reduced putative AMP complement (6.95 ±0.01 AMP-encoding genes per genome; 0.06 ± 0.01% of PCGs encoding AMPs) in comparison to Clade 9/V species. This reduced AMP complement was particularly evident within the Filarioidea species which may suggest that there has been a loss of putative AMP genes from filarial nematodes. In line with the more basal nematodes in Clade 2/I, reduced profiles of metabolic and neuropeptide pathway components are also observed in filarial nematodes [59,61]. Filarial nematodes typically reside in the circulatory system and subcutaneous tissues of the definitive host and may, therefore, be less reliant on AMPs due to the more sterile host niches they occupy. Additionally, many filarial nematodes including *Brugia malayi*, *Onchocerca volvulus* and *Wuchereria bancrofti* share well characterised endosymbiotic relationships with *Wolbachia* which are critical for key aspects of nematode biology [62]. It is possible that *Wolbachia* provides a level of protection for filarial parasites such that the nematodes require fewer endogenous AMPs. Alternatively, the reduced putative AMP profiles in Filarids could help shape a favourable environment for *Wolbachia* growth.

Clade 12/IV species, which include the PPNs, also possess a reduced putative AMP profile (27.47± 4.54 AMP-encoding genes per genome; 0.09± 0.01% of PCGs encoding AMPs) in comparison to Clade 9/V nematodes. Notably, the only PPN in this study that is not a Clade 12/IV species, *Bursaphelenchus xylophilus* (Clade 10/IV), possesses 61 putative AMP-encoding genes (0.34% relative abundance), exhibiting a profile much more in line with Clade 10/IV species. As noted for Clade 2/I nematodes there is also potential that there are currently undiscovered AMP groups within Clade 12/IV species with diverse motifs that are not identifiable via the methods employed here. This highlights the need to expand AMP studies beyond *C*. *elegans* and *A*. *suum* to discover more distinct AMP groups.

### Cecropin genes are entirely restricted to Ascaridoidea species

The pan-phylum data presented here provides evidence to support the restriction of putative Cecropin-encoding genes to Ascarid species; these data also report the first identification of Cecropin-encoding genes from *Parascaris* spp and confirm the recent identification of Cecropins from *Anisakis* species [63]. The restriction of Cecropins to Ascaridoidea suggests Ascarid-specific roles that require further characterisation. As noted above, Cecropins were originally isolated from the porcine intestinal environment [13]. Despite this, the effect of these peptides on host microbiota has yet to be characterised.

**Fig 3:**
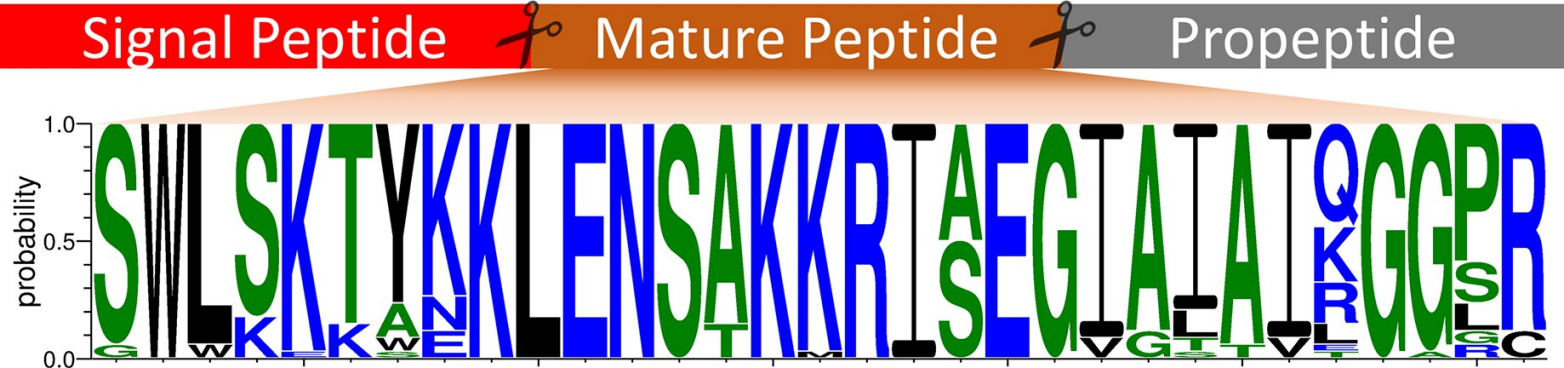
Ascaridoidea-restricted Cecropins display significant amino acid conservation. Weblogo generated using 34 putative cecropin peptide sequences (4 peptides excluded due to gene mispredictions; GS_20806, TCNE_0000065801, Tcan_02070, GS_06967) using WebLogo3 [https://weblogo.threeplusone.com/] [65–66], coloured according to amino acid hydrophobicity (hydrophilic = blue, neutral = green, hydrophobic = black). Amino acid letter height reflects the probability of conservation at a specific location. Signal peptide, mature peptide and propeptide indicated.

Putative Cecropins have not undergone the same gene expansion observed in other AMP groups (4.75 ± 0.88 genes per genome). The Ascarid Cecropins appear to be highly conserved suggesting that these peptides have similar antimicrobial activities (Fig 3). Indeed, chemically synthesised *A*. *suum* Cecropin P1-P4 have similar activity profiles against bacteria and fungi [28]. However, minor differences in amino acids can significantly change the antimicrobial activity of these peptides; for example, a single amino acid substitution of a Proline residue for Isoleucine residue at position 22 of *A*. *suum* Cecropin P1 significantly reduces antibacterial and membrane permeabilising activity [64]. Recently, Cecropin peptides from *Anisakis* species were shown to have varying antibacterial activities against five bacterial species as well as differences in the transcript expression of Cecropin genes according to the host animal infected [63]. These studies demonstrate the need to characterise AMPs from the same groups across different species.

### Diapausin genes are restricted to specific free-living soil nematodes

Nematode Diapausin-encoding genes were originally identified from *Pristionchus pacificus* [18]. The pan-phylum analyses performed here reveals that Diapausins are restricted to Clades 9/V and 11/IV in soil-dwelling FLNs, primarily the Clade 9/V Diplogasteroidea which includes *Pristionchus* spp. Beyond the Diplogasteroidea, two Rhabditoidea, *Mesorhabditis belari* and *Oscheius tipulae*, also encode Diapausins while the closely related *Caenorhabditis* genera do not. The absence of Diapausin-encoding genes in *C*. *elegans* is interesting given the similar environmental niche occupied by these species, highlighting the importance of AMP research beyond *C*. *elegans*. Diapausin-encoding genes were also identified in *Acrobeloides nanus*, the only Clade 11/IV species in this study; this is an intriguing observation from an evolutionary perspective.

It has been suggested that *P*. *pacificus* Diapausin-encoding genes have been acquired through horizontal gene transfer from beetles with which they share phoretic/necromenic associations [67]. This theory is supported by the presence of a Diapausin-like sequences in an insect iridovirus implicating the virus as the vector for gene transfer [22]. Phylogenetic analyses did not provide any further insight into these relationships or gene evolution primarily due to poor resolution as a consequence of the rapid evolution of AMP-encoding genes. More work is needed to explore the distribution of Diapausin-like genes across diverse animal taxa in order to confirm this hypothesis.

**Fig 4:**
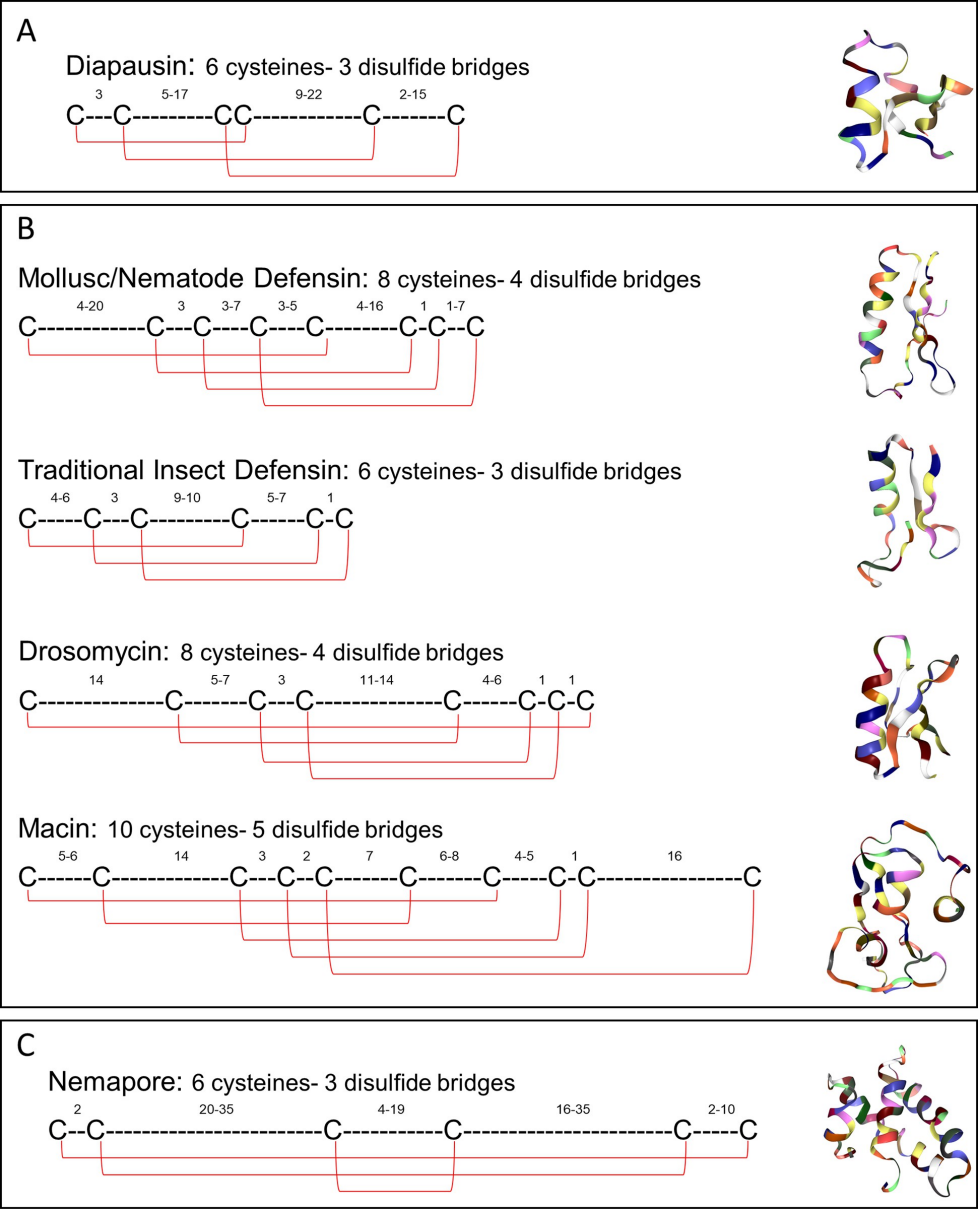
Cysteine organisation of the nematode cysteine-stabilised AMP groups enables delineation of AMP subgroups. A: The putative Diapausin cysteine organisation. Peptide group 3D structure example: *Gastrophysa atrocyanea* Diapause-specific peptide (RCSB: 2E2F). B: Organisation of cysteine residues in the putative CSαβ members. Peptide group 3D structure examples: Mollusc/Nematode Defensin (*Ascaris suum* ASABF-α; RCSB:2D56), Traditional Insect Defensin (*Sarcophaga peregrina* Sapecin; RCSB: 1L4V), Drosomycin (*Drosophila melanogaster* Drosomycin; RCSB: 1MYN), Macin (*Hirudo medicinalis* Theromacin; RCSB:2LN8). C: The putative Nemapore cysteine organisation. Peptide group 3D structure example: *Caenorhabditis elegans* Caenopore-5 (RCSB:2JS9). ‘C’ indicates a Cysteine residue. ‘-’ indicates any other amino acid. Subscript numbers indicate the range in number of amino acids between each cysteine residue. Red lines indicate putative disulphide bonds between cysteine residues. Peptide group 3D structures obtained from Research Collaboratory for Structural Bioinformatics Protein Data Bank (RCSB PDB) [https://www.rcsb.org/] [68].

In total 116 putative Diapausin-encoding genes were identified across phylum Nematoda with an average of 8.92 ±1.62 genes per genome. Diapausins possess a cysteine motif that consists of six cysteine residues which form three disulfide bonds (see Fig 4A). The position of these cysteines is conserved compared to arthropod Diapausins, suggesting conservation in Diapausin tertiary structure. Interestingly, some of the Diapausin-encoding genes identified in this study possessed two complete cysteine Diapausin motifs (e.g. ACRNAN_scaffold769.g15805; 12 cysteine residues). So far, Diapausin-encoding genes have only been reported to possess a single Diapausin cysteine motif and thus it is unclear whether the genes identified here contain multiple domains from errors in gene annotation or whether they represent a novel type of Diapausin-encoding gene, where multiple peptides could be cleaved from a single gene precursor. Beyond the nematode Diapausin cysteine motif there is significant amino acid variation that may result in different antimicrobial activity profiles between individual peptides. While Diapausins in beetles are potent antifungal agents which play a role in diapause [22], the role of Diapausins in nematodes has yet to be established.

### Classification of nematode CSαβ peptides reveals complexity in subgroup distribution

CSαβ peptides are a complex group of peptides which share a tertiary structure comprising an α-helix and two antiparallel β-sheets stabilised by three disulfide bonds [69]. CSαβ peptides can be classified into multiple subgroups of AMPs displaying differences in cysteine organisation. The putative CSαβ peptides identified in this study have been classified based on the presence of a shared cysteine organisation but this does not indicate that they share the same evolutionary origin. 533 putative CSαβ-encoding genes (3.98 ±0.50 genes per genome) were identified in this study and were broadly distributed across phylum Nematoda, with representatives in every nematode clade (see Fig 2). Putative CSαβ-encoding genes are more restricted in Clades 2/I (only *R*. *culicivorax*) and 8/III (only Ascaridoidea and Oxyuroidea spp.), which is consistent with a previous study [19]. In contrast, CSαβ-encoding genes are more widespread in Clades 9/V, 10/IV and 12/IV (see Fig 2); note that two CSαβ-encoding genes, previously identified from *P*. *trichosuri* EST datasets [19], were not identified here.

**Fig 5:**
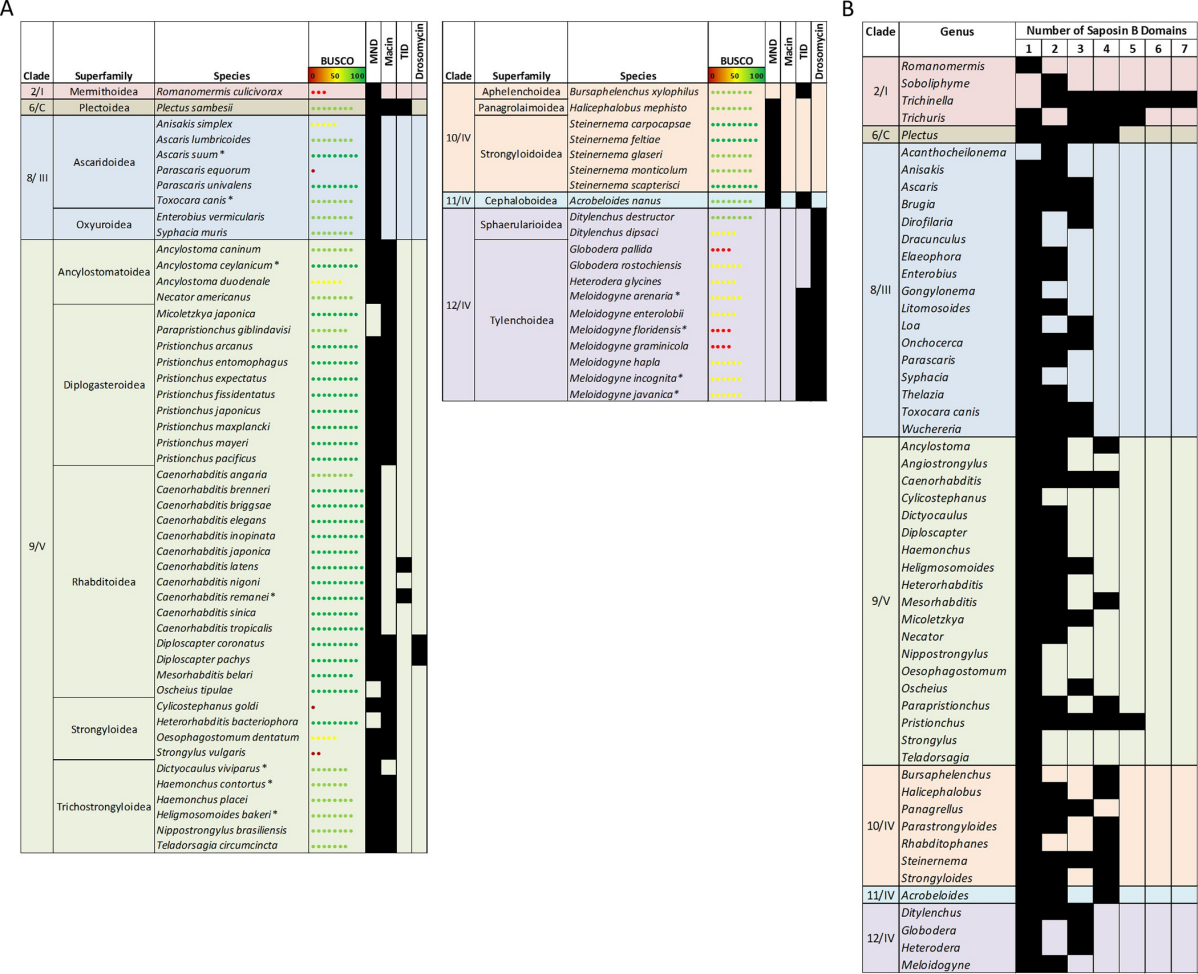
The distribution of putative CSαβ subgroups and putative multi-domain Nemapores reveal additional AMP group diversity across nematode clades. A: The distribution of four putative CSαβ subgroups [Mollusc/Nematode Defensin (MND), Macin, Traditional Insect Defensin (TID), Drosomycin] in CSαβ-containing nematode species. B: The distribution of putative multi-domain Nemapore-encoding genes across nematode genera. Nematode species/genera are organised by clade [47–48] and superfamily designation (NCBI taxonomy browser; https://www.ncbi.nlm.nih.gov/taxonomy). Black shading indicates the presence of at least one putative AMP gene identified via HMM, BLASTp and tBLASTn analyses. BUSCO complete scores were obtained from WormBase ParaSite V14 [https://parasite.wormbase.org/index.html] [34]. Note that species which do not possess CSαβ encoding genes are not included in A.

CSαβ-encoding genes identified in this study were classified into four subgroups based on the cysteine reference array reported previously [24]: Mollusc/Nematode Defensins (MND), Traditional Insect Defensins (TIDs), Macins and Drosomycins. The distribution of these subgroups was variable across phylum Nematoda (see Fig 5A). The MND subgroup, previously known as Antibacterial Factors [ABFs; 23], was the most widely distributed, identified in almost all clades examined (see Fig 5A). MNDs are characterised by a cysteine motif consisting of eight cysteine residues which form four putative disulfide bonds (see Fig 4B). In line with the Diapausins, high amino acid variability exists between the key MND cysteine residues, which may contribute to diversity in antimicrobial activity. MND antimicrobial activity has only been investigated in *A*. *suum* ASABF-α and *C*. *elegans* ABF-1 and -2 which possess potent antibacterial and antifungal activity [14,70]. Putative MNDs were identified in every nematode clade except for Clade 12/IV, suggesting that they may have been acquired by an ancestral nematode and subsequently lost in the genera now lacking MND-encoding genes (Clade 2/I *Trichinella* and *Trichuris* spp., Clade 8/III filarial species, and all Clade 12/IV species). The reduced distribution of the MND subgroup is consistent with the broadly reduced AMP complement in Clade 2/I and 8/III suggesting a negative selective pressure for AMP-encoding genes in these clades, whereas Clade 12/IV displays other CSαβ subgroups such that the lack of MNDs could be due to functional redundancy.

A MND member with only six cysteine residues and a rearranged disulfide bonding pattern, known as ASABF-6Cys-α, was previously identified in *A*. *suum* [71]. Previous analysis of EST and draft genomic datasets identified homologs of ASABF-6Cys-α beyond Ascaridida in *Ancylostoma ceylanicum*, *Caenorhabditis briggsae*, *Caenorhabditis remanei* and *Meloidogyne hapla* [19]. However, this study has revealed that the *A*. *ceylanicum*, *C*. *briggsae and C*. *remanei* sequences are, in fact, not homologs of ASABF-6Cys-α but are traditional MND peptides which were previously incomplete. The *M*. *hapla* sequences were not included in this analysis as they lack key MND motifs. In this study, a single putative ortholog of *A*. *suum* ASABF-6Cys-α was identified in *Parascaris univalens* supporting the original hypothesis that the 6-cysteine variation in the MND sequence is only present in nematodes in the Ascaridida [71].

Nematode Macin-encoding genes have been previously reported [72] however this study provides the most comprehensive profile of these genes across phylum Nematoda. Putative Macin-encoding genes were identified in Clades 6/C and 9/V. Within Clade 9/V, Macin-encoding genes are found in all species except all *Caenorhabditis* spp., *Dictyocaulus viviparus* and both *Angiostrongylus* spp. Notably, putative Macin-encoding genes are found in parasites of health and economic importance including *H*. *contortus*, *N*. *americanus* and *T*. *circumcincta*. The absence of putative Macin-encoding genes from the *Caenorhabditis* genera could suggest that they have been lost in these species while they have been retained in other free-living Clade 9/V nematodes. The reason for this loss is unclear but reemphasises the need to conduct AMP research beyond *C*. *elegans*.

Macins from other invertebrates possess antibacterial and antifungal activities and can promote regeneration of neuronal tissue in annelids [73], however nematode Macins have not been functionally characterised. In other invertebrate phyla, Macin peptides possess eight to twelve cysteine residues [72] whereas nematodes appear to encode Macins with ten cysteine residues (see Fig 4B). Theromacin from the annelid *Hirudo medicinalis*, which exhibits activity against gram positive bacteria and promotes neuron regeneration, also possesses ten cysteine residues suggesting that nematode Macins may possess similar functional profiles.

Drosomycin peptides possess eight cysteine residues which form four putative disulfide bonds to produce a tertiary structure that is distinct from the MNDs (see Fig 4B). Putative Drosomycin-encoding genes were previously identified in free-living *Panagrolaimus superbus* (Clade 10/IV) EST datasets [19]. In this study, there were no *Panagrolaimus* genomes included in the nematode database however, the addition of four *Panagrolaimus* genomes in a subsequent WormBase ParaSite update (Version 15, October 2020) allowed the confirmation of Drosomycin genes in the *Panagrolaimus* genera.

Putative Drosomycin-encoding genes were also identified in Clade 12/IV species and two Clade 9/V species (*Diploscapter coronatus* and *Diploscapter pachys*). It is unclear whether other Clade 10/IV species have lost these genes or whether the *Panagrolaimus* species acquired them independently. Nematode Drosomycin peptides have yet to be functionally characterised however insect Drosomycin peptides display potent antifungal activity against filamentous fungi [74]. Note that cysteine-rich nematode peptides have previously been designated as Drosomycin peptides but have since been reclassified as TID subgroup members based on their cysteine motif positioning [24].

Traditional Insect Defensin peptides are characterised by the typical insect Defensin cysteine array where six cysteines form three disulfide bonds (see Fig 4B). Putative TIDs display a more limited distribution across phylum Nematoda (28 TID-encoding genes in 12 nematode species). Intriguingly, the 12 nematode species that possess putative TID-encoding genes are phylogenetically distinct FLN and PPN species that span five nematode clades; this raises questions about the evolutionary history of these genes. The TID subgroup members appear to have functionally diversified such that each peptide has a specific activity range. For example, *Caenorhabditis remanei* Cremycin-5 possesses antifungal activity whereas Cremycin-15 lacks antifungal activity but is bioactive against gram positive bacteria [75].

### Nemapores are the most widely distributed AMP group, and can be delineated based on Saposin domain characteristics

Nemapores are cysteine-stabilised AMPs found in a wide range of nematode species. They are members of the SAPosin-LIke Protein (SAPLIP) superfamily which includes other membrane-interacting proteins such as metallophosphoesterases and cell proliferator regulators [36]. Nemapores are characterised by a signal peptide sequence followed by at least one Saposin B domain. Saposin B domains comprise of six cysteine residues in a specific arrangement (see Fig 4C). This study updates previous analyses which indicate that Nemapores are the most widely distributed AMP group in phylum Nematoda [see Fig 2; 19]. Indeed, at least one putative Nemapore-encoding gene (~15.34 ±1.38 genes per genome) was identified from every species analysed, suggesting that Nemapores may be a key component of nematode innate immunity. Putative Nemapore-encoding genes, which were previously reported as absent in *N*. *americanus*, *Strongyloides stercoralis* and *Meloidogyne incognita* [19], were identified in this study reemphasising the value in utilising updated genomic datasets.

Despite broad representation across the phylum, the numbers of putative Nemapore-encoding genes varies considerably across clades and species. Clades 6/C, 9/V and 10/IV appear to be enriched for Nemapore-encoding genes; for example, *Plectus sambesii* has >100 putative Nemapore-encoding genes. The elevated number of putative Nemapore-encoding genes in Clade 10/IV, highlights that Nemapores may play a key role in Clade 10/IV nematodes. Additionally, *C*. *elegans* Nemapores have been implicated in nutrition, where knockdown of *Ce-spp-5* resulted in a diminished capability to kill ingested *Escherichia coli* resulting in impeded development [76].

There is also variation in the number of Saposin B domains encoded by Nemapore genes across nematode genera (see Fig 5B). Previous analyses indicate that Nemapores containing three and four Saposin domains were restricted to species from Clades 2/I and 9/V [19]. Data generated in this study, however, show that putative multi-domain Nemapores are more widespread where Nemapores with three or more Saposin B domains are represented in all clades. Indeed, *Trichinella* spp. appear to be enriched for multi-domain Nemapores with some genes containing seven Saposin B domains; multi-domain Nemapore genes have yet to be functionally characterised. In the amoeba *Naegleria fowleri* multiple bioactive Saposin peptides are processed from larger multipeptide precursor sequences [77]. It is also possible that nematode multi-domain Nemapores are post translationally cleaved to produce multiple bioactive single domain Nemapores however previous attempts to unravel the processing of a four Saposin domain protein from *Trichinella spiralis* were unable to resolve protein cleavage [78].

### Enriched numbers of putative GRSPs are not restricted to the *Caenorhabditis* genera

This study provides the first pan-phylum profile of putative GRSP-encoding genes across phylum Nematoda; previously GRSPs had only been profiled in *C*. *elegans* and *C*. *briggsae* where they were found to be enriched relative to several other model organisms [20]. The data presented here demonstrates that GRSPs appear to be the most abundant AMP group in phylum Nematoda (3145 genes; 23.47 ±2.26 genes per genome) with Clades 9/V and 10/IV exhibiting enriched profiles. The enriched number of putative GRSPs extends beyond *Caenorhabditis* genera (see Fig 2), where GRSP-encoding genes are absent from only two nematode species [*Dracunculus medinensis* and *Litomosoides sigmodontis* (Clade 8/III)] which suggests importance to nematode biology. Despite sharing sequence similarity, it is not clear if the putative GRSPs identified here share an evolutionary origin. It is important to note that the highly repetitive glycine rich nature of the GRSP group makes establishing evolutionary relationships challenging and further research is required to understand the evolution of GRSPs across phylum Nematoda. Functional characterisation of GRSPs has been restricted to *C*. *elegans* where their expression has been profiled post microbial infection [20], and antibacterial and antifungal assays reveal antimicrobial properties that do not involve membrane permeabilisation [17,79]. Further work is crucial to confirm that the genes identified here do encode bioactive AMPs. In addition, it is interesting to note that a number of Neuropeptide-Like Proteins (NLPs) have been classified as GRSPs (NLP-10, NLP-12, NLP-21 and NLP-24-34), highlighting a need to unravel the role of these peptides.

### Publicly available transcriptome and proteomic datasets support the prioritisation of nematode putative AMPs

**Fig 6:**
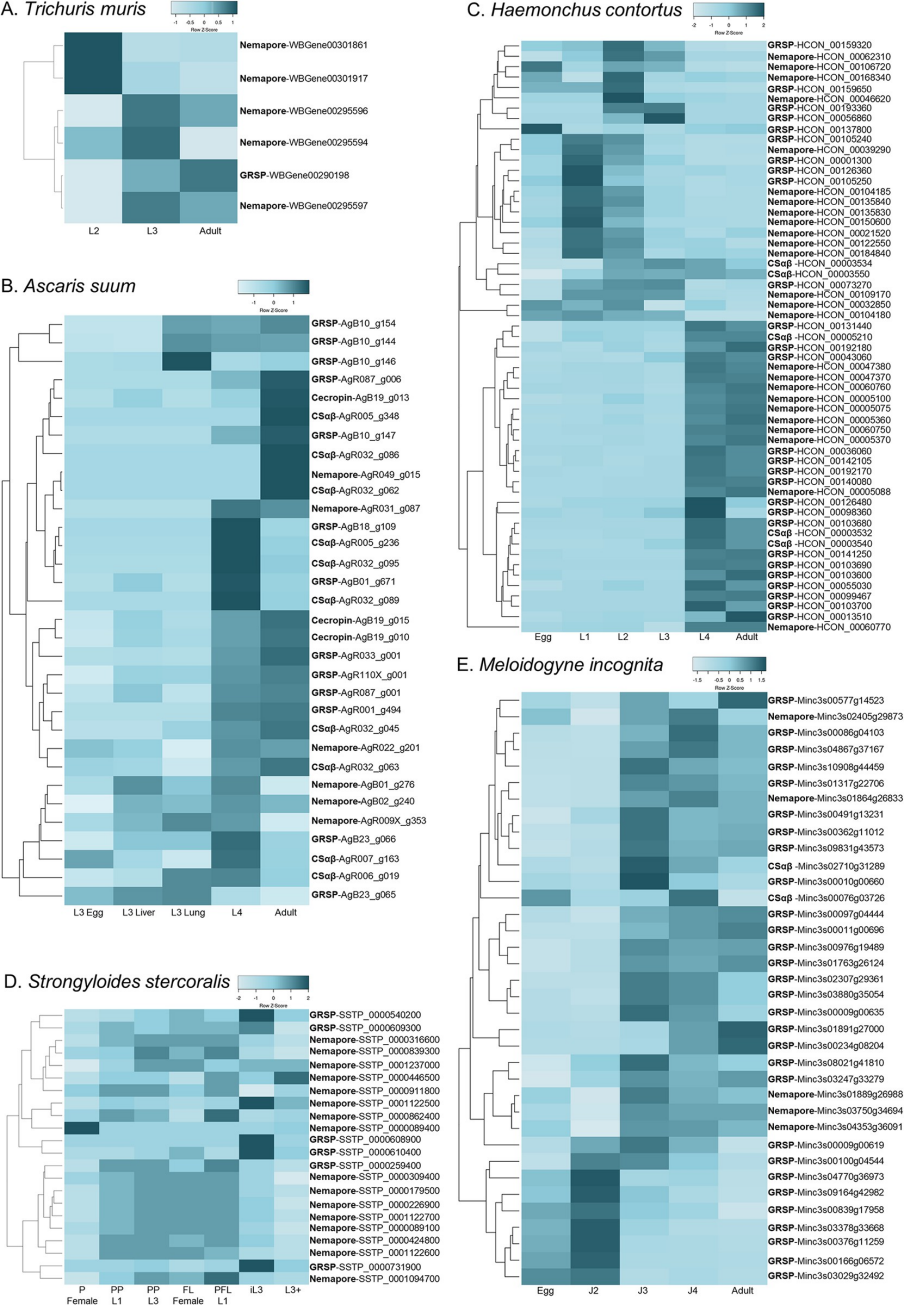
Summary of transcriptomic data. Life stage specific heatmaps of AMP expression for AMP genes from A: *Trichuris muris* (6 genes), B: *Ascaris suum* (32 genes), C: *Haemonchus contortus* (58 genes), D: *Strongyloides stercoralis* (22 genes), E: *Meloidogyne incognita* (36 genes). Heatmaps generated using Heatmapper [http://www.heatmapper.ca/] [44]. Expression is shown in Z scores and scaled by row. Genes are clustered using Average Linkage and Euclidean distance method. Darker shades represent higher expression and lighter shades represent lower expression.

To assess the biological relevance of putative AMPs across nematode life stages, publicly available life stage specific RNAseq datasets were examined for 15 species (10 APN, 1 EPN, 3 PPN, 1 FLN). Of the putative AMP genes identified >85% were expressed (TPM >2) in at least one life stage (see Fig 1E). Notably, despite significant variation in the total number of putative AMP genes encoded within individual nematode species, this did not translate to major variability in the proportion of total AMP genes expressed. For example, in *Trichuris muris* six of the seven (86%) identified AMP genes were expressed whereas in *P*. *pacificus* 88 of the 102 (86%) identified AMP genes were expressed. There appear to be differences in the expression of putative AMP genes from the same AMP group such that no AMP group is consistently expressed in a specific life stage in the five parasitic species examined in more detail (see Fig 6). This suggests that closely related putative AMPs may have functionally diversified and reinforces the need to further characterise these putative AMPs in other species. Notably, in *A*. *suum* and *H*. *contortus* there is clear elevation in the expression of putative AMP genes in life stages which are exposed to the host gastrointestinal system further supporting that these genes may play a role in nematode innate immunity.

To validate the prediction of the putative AMP-encoding genes identified through *in-silico* analyses in this study, previously published proteomic/peptidomic datasets (see Table 2) were analysed for the presence of AMP candidates in the excretory/secretory products (ESP) and biofluids of *A*. *suum* (ESP, uterine fluid and pseudocoelomic cavity fluid [80,81]), *H*. *contortus* (L3, L4 and adult ESP [82]), *Steinernema feltiae* (infective juvenile ESP [83]) and *Trichuris suis* (adult ESP [84]). Twenty-six AMP candidates were detected (see Table 2) supporting their potential biological relevance in nematodes. Interestingly, no putative AMPs were detected in the pseudocoelomic cavity fluid using proteomics approaches [80] in contrast to five putative AMPs that were detected using peptidomics approaches [81]. This demonstrates that a peptidomic workflow may be more appropriate for AMP discovery. In addition, the peptides identified here (see Table 2), through analysis of existing proteomics datasets, were not originally identified as putative AMPs as the peptides were uncharacterised. The comprehensive BLAST/HMM approach employed here enabled the retrospective interrogation of all putative AMP peptides in these datasets underscoring the value of this study.

**Table 2:** Overview of AMP candidates detected in proteomic [80,82–83,84] or peptidomic [81] studies.

Species	Source	Gene ID	AMP Group
*Ascaris suum*	ESP [80]	GS_10369 (ASABF-β)	CSαβ
		GS_05608 (ASABF-α)	CSαβ
		GS_08337 (ASABF-γ)	CSαβ
	UF [80]	GS_19916	CSαβ
	PCF [81]	AgB19_g015 (Cecropin P1)	Cecropin
		GS_14502 (Cecropin P2)	Cecropin
		AgB19_g010 (Cecropin P3)	Cecropin
		AgR032_g063 (ASABF-α)	CSαβ
		AgR032_g045 (ASABF-6Cys-α)	CSαβ
*Haemonchus contortus*	L3 ESP [82]	HCON_00062310	Nemapore
	L3, L4 & Adult ESP [82]	HCON_00047370	Nemapore
	L4 & Adult ESP [82]	HCON_00003550	CSαβ
		HCON_00104180	Nemapore
		HCON_00060770	Nemapore
		HCON_00060760	Nemapore
	Adult ESP [82]	HCON_00005370	Nemapore
		HCON_00005360	Nemapore
		HCON_00005100	Nemapore
*Steinernema feltiae*	Activated infective juvenile ESP [83]	L889_g29545	Nemapore
		L889_g13905	Nemapore
		L889_g31036	Nemapore
		L889_g31037	Nemapore
		L889_g17551	Nemapore
		L889_g20297	Nemapore
		L889_g6015	Nemapore
*Trichuris suis*	Adult ESP [84]	D918_09288	Nemapore

ESP, excretory/secretory products; PCF, pseudocoelomic fluid; UF, uterine fluid.

### Limitations of BLAST/HMM-driven AMP discovery in nematodes

Antimicrobial peptide identification through *in silico* studies is useful for providing peptide libraries which can enable prioritisation of putative AMP targets for functional analyses. However, there are limitations associated with *in-silico* AMP discovery. The putative AMPs identified in this study were discovered through a sequence similarity pipeline and require functional characterisation in order to confirm their putative antimicrobial activities and determine their putative role(s) in nematode biology.

Genome and transcriptome studies also possess inherent caveats that make extrapolation of the data challenging. For example, in this study there are significant differences in the quality of the genome datasets where some chromosomal level assemblies (e.g. *Strongyloides ratti*) are available while others remain at contig level (e.g. *M*. *hapla*). The quality of genome datasets was considered through the integration of BUSCO complete scores for each genome [see Fig 2 and S2 File] [85]. It is important to note that BUSCO datasets are primarily derived from free-living organisms and as a result, gene losses as a result of parasitism may incorrectly give the impression of poorer genome quality based on BUSCO score. Despite this, there appears to be an association between the number of AMPs genes identified and genome quality as denoted by BUSCO score; for example, 7 AMP genes in *Parascaris equorum* (BUSCO complete score of 5) relative to 31 AMP genes in the closely related *Parascaris univalens* (BUSCO complete score of 90.9). For many lower quality genomes, there were fewer than expected AMPs identified and many of those identified appeared incomplete, either predicted as part of longer proteins or missing key sequence motifs. As nematode genome assemblies and annotations are improved, more AMP genes will be uncovered to reveal a more comprehensive nematode antimicrobial peptidome.

The BLAST/HMM-derived approach used in this study to identify AMPs was driven by query sequences primarily derived from *C*. *elegans* and *A*. *suum*, such that the HMM models are likely to be more biased towards the identification of *Caenorhabditis*- or *Ascaris*-like AMPs. Consequently, it is possible that AMPs which have evolved rapidly in distantly related species may be overlooked using this approach, underscoring the need to develop alternative strategies for *in silico* AMP discovery. Indeed, it is difficult to assess the significance of the apparent reduced AMP profiles in Clades 2/I, 8/III and 12/IV until comprehensive AMP discovery approaches have been deployed across phylum Nematoda. Verification of AMPs in nematode tissues and biofluids using peptidomics technologies [81] will seed follow-on functional biology.

## Conclusions

This study provides an *in silico* comprehensive genome-level pan-phylum profile of five groups of putative nematode AMP-encoding genes which will inform future analyses into the function of these putative AMPs. These data demonstrate that nematodes appear to have a rich AMP complement that includes 5887 putative AMP-encoding genes in 109 species, ~90% of which are novel. The pan-phylum approach has revealed that APNs and PPNs possess fewer putative AMP-encoding genes than FLNs suggesting that parasitic nematodes may possess a more specific AMP arsenal tailored towards the host environments they inhabit. Major differences in the distribution and abundance of putative AMP groups were evident across the phylum where Clade 2/I, 8/III and 12/IV species possess fewer putative AMP-encoding genes, while Clade 9/V and 10/IV species have expanded AMP repertoires. Two AMP groups, Cecropins and Diapausins, appear to be restricted to Ascarids and FLNs respectively whereas CSαβ, Nemapores and GRSPs were more widely distributed across the phylum. This study provides a database of putative AMPs in a range of economically relevant nematode species that should be functionally characterised to unravel the importance of AMPs to parasite biology.

## Supporting information

S1 Fig Coloured CLANS sequence similarity network for nematode Cecropins, Diapausins, CSαβs and Nemapores. Network was generated after 10,000 rounds of clustering from all-against all protein BLAST searches between all complete sequences (E-value limit = 1e-4). Sequences are coloured according to group or subgroup designation.
(TIF)

S1 File AMP query sequences used for construction of AMP group HMMs. (XLSX)

S2 File Genomic and transcriptomic datasets used in this study. (XLSX)

S3 File AMP gene IDs identified from 134 nematode predicted protein datasets. (XLSX)

S4 File Number and relative abundance of putative AMP genes identified in this study. (XLSX)
