# Supplementary figures and images for: Exploring the antimicrobial peptidome of nematodes through phylum-spanning in silico analyses highlights novel opportunities for pathogen control
Source: PLoS Negl Trop Dis. 2023 Sep 6;17(9):e0011618. doi: 10.1371/journal.pntd.0011618 (PMC10506718; doi:10.1371/journal.pntd.0011618)

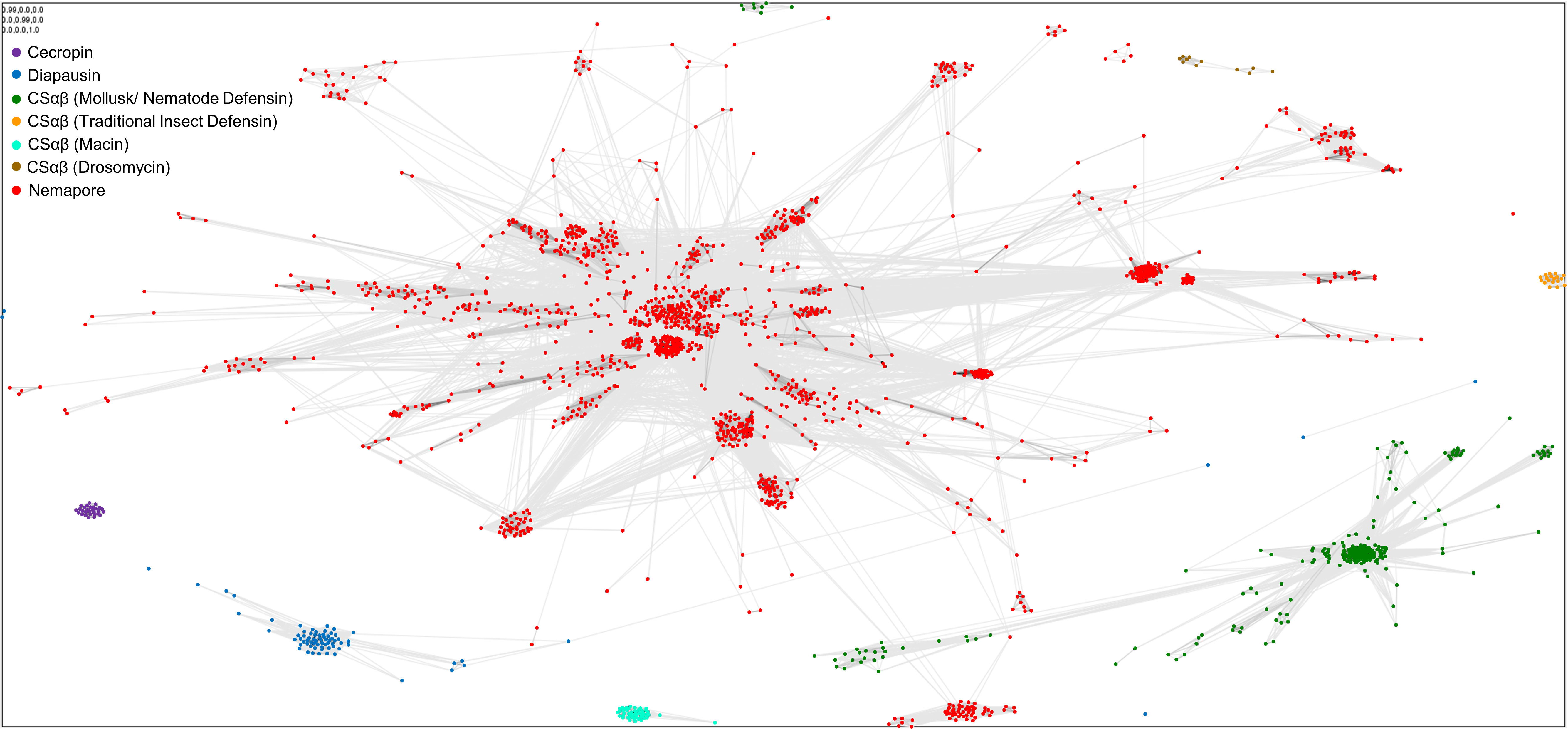

Supplement: S1 Fig — Network was generated after 10,000 rounds of clustering from all-against all protein BLAST searches between all complete sequences (E-value limit = 1e-4). Sequences are coloured according to group or subgroup designation. (TIF) [file pntd.0011618.s001.tif]
